# Cytokeratin-Positive Osteosarcoma Simulating Sarcomatoid Metastatic Carcinoma

**DOI:** 10.1155/2020/3761015

**Published:** 2020-02-04

**Authors:** Hamza Murtaza, Abdul Rehman Arain, Afshin Anoushiravani, Sean Thadani, Gustavo de la Roza, Rana Naous, Timothy A. Damron

**Affiliations:** ^1^Albany Medical Center, Albany NY, USA; ^2^St. Matthew's University, West Bay, Cayman Islands; ^3^Department of Pathology, Upstate Medical University, Syracuse NY, USA; ^4^Department of Orthopedic Surgery, Upstate Medical University, Syracuse NY, USA

## Abstract

Osteosarcoma, the most common primary malignant bone tumor, rarely stains positive for epithelial markers such as cytokeratin on immunohistochemical analysis. We describe a 52-year-old man with a destructive distal femoral metaphyseal lesion who was initially treated for metastatic sarcomatoid carcinoma based on extensive radiographic and histopathologic evaluation. Ultimately, wide resection of the distal femur was performed, revealing high-grade conventional osteosarcoma with intense and diffuse cytokeratin positivity. Such immunohistochemical staining in osteosarcoma is rare, making it difficult to distinguish cytokeratin-positive osteosarcoma from metastatic carcinoma. The presence of a cytokeratin-positive bone neoplasm with malignant osteoid formation on histological studies as well as integration with clinical and radiologic data can help confirm osteosarcoma as the ultimate diagnosis.

## 1. Introduction

Osteosarcoma is the most frequent primary malignant tumor of the bone [1]. It is very rare for osteosarcoma to show intense and diffuse positivity for epithelial markers such as cytokeratin on immunohistochemical analysis [2, 3]. Few reports have previously described differentiation in osteosarcomas that resemble metastatic disease [4–12]. This presents a unique diagnostic challenge, as metastatic carcinoma must be distinguished from osteosarcoma since the treatment and management differs. The following case describes a patient with high-grade conventional osteosarcoma of the distal femur showing epithelial differentiation on immunohistochemistry as indicated by strong and diffuse cytokeratin positivity.

## 2. Case Presentation

A 52-year-old Caucasian male, with no prior history of malignancy or bone lesions, presented with a 9-month history of atraumatic, aching, moderate, intermittent pain in the right knee and lower leg with insidious onset, radiating distally and awakening the patient from sleep at night, exacerbated by weight-bearing, lifting, activity, movement, and local pressure. No constitutional symptoms were reported. Imaging ordered by his primary care physician (PCP) revealed a permeative destructive process involving the distal femoral metaphysis, leading to orthopedic referral.

Physical exam showed slight right-sided antalgic gait and tenderness to palpation of the right knee lateral joint line, distal lateral femur, and lateral femoral condyle. Right knee active range of motion was 5-110 degrees. Neurovascular status was normal.

Blood work showed normal complete blood count, a metabolic panel including lactate dehydrogenase, and serum protein electrophoresis. Prostate-specific antigen levels were within normal limits (<4.0 ng/mL).

**Figure 1:**
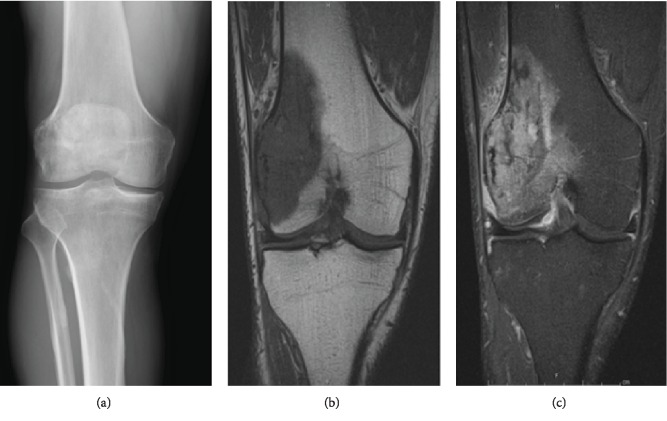
(a) X-ray of the involved distal femur shows a mixed lucent sclerotic lesion with permeative borders involving the lateral distal femoral metaphysis. (b) MRI shows a bone lesion replacing the marrow in the LFC and distal metadiaphyseal junction with dark heterogeneous signal on T1W and brighter signal intensity on T2W sequences. (c) Perilesional edema is seen on the T2W sequences both in the intraosseous and periosteal regions. There was no definite associated STM. Maximal dimensions were just under 8 cm.

**Figure 2:**
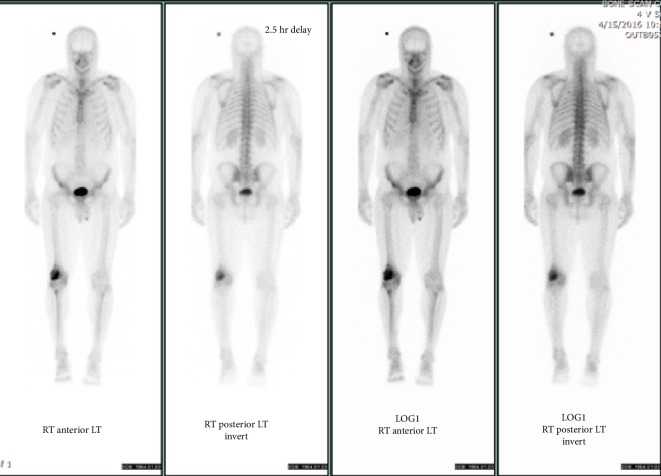
Whole body Tc-99m bone scan showed isolated increased activity in the right distal femur.

Radiographic workup consisted of plain X-rays of the knee (Figure 1(a)) as well as magnetic resonance imaging (Figures 1(b) and 1(c)). Staging workup consisted of chest/abdomen/pelvis computed tomography and Tc-99m total body bone scan (Figure 2). Based on these studies, the lesion appeared to be a solitary bone lesion without any disease elsewhere.

**Figure 3:**
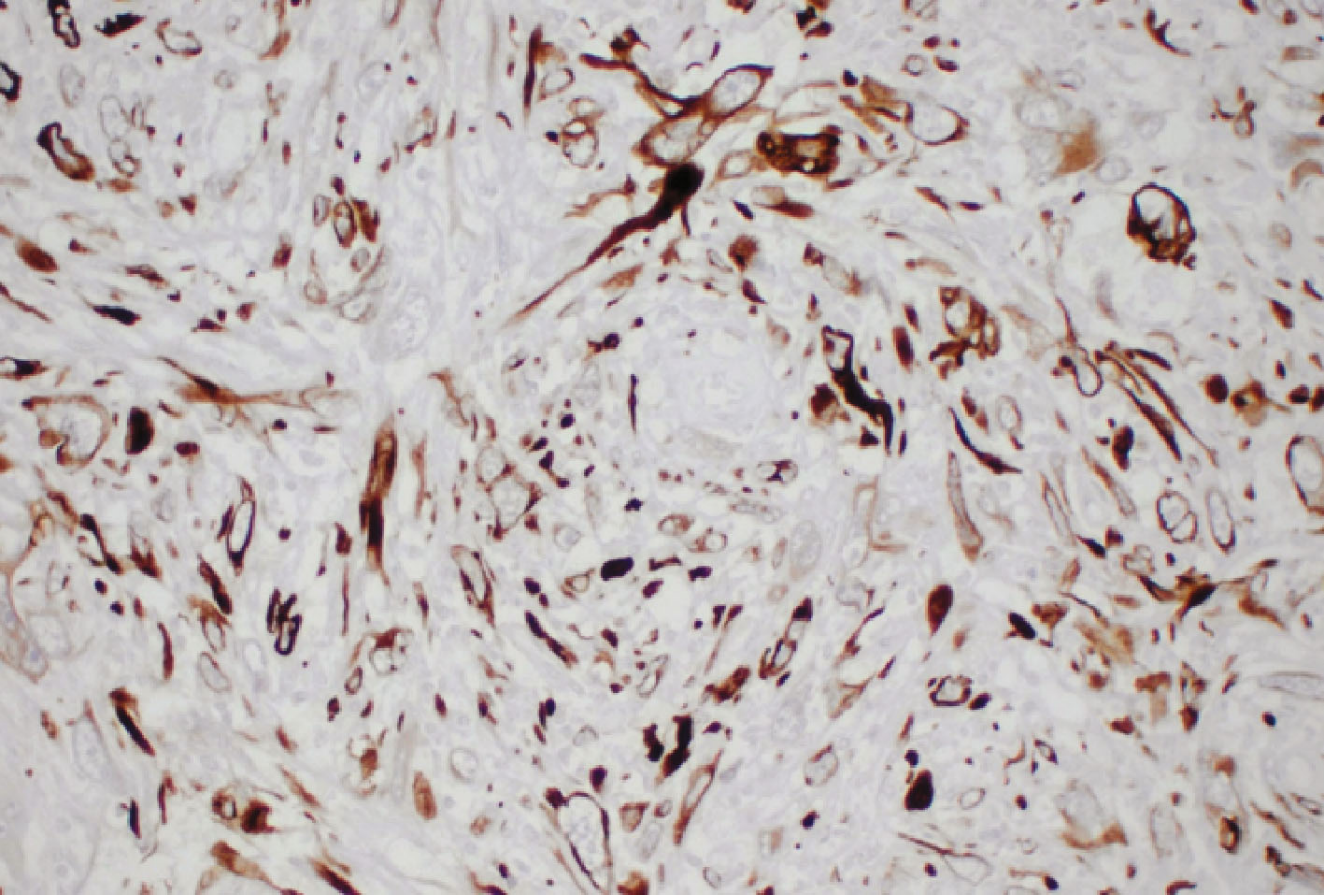
Malignant cells with strong cytoplasmic reactivity with cytokeratin CAM 5.2 (H&E stain, 20x).

Occult solitary metastatic carcinoma, primary bone lymphoma, and primary bone sarcoma remained highest among the list of differential diagnostic considerations. An open biopsy was performed yielding a frozen section that was inconclusive. Permanent sections showed a malignant spindle cell neoplasm with marked cellular pleomorphism and extensive necrosis infiltrating lamellar bone. There were bone necrosis and remodeling, but no clear evidence of osteoid matrix or bone formation. The morphological differential diagnosis mainly included metastatic sarcomatoid carcinoma and osteosarcoma. Immunohistochemistry showed that the tumor was strongly and diffusely positive for vimentin and cytokeratin CAM 5.2 (Figure 3), focally and weakly positive for GATA-3 and cytokeratin AE1/AE3. No reactivity was seen with epithelial membrane antigen (EMA), PAX-8, RCC, desmin, smooth muscle actin, myogenin, S-100, SOX-10, MART-1, TTF-1, CD34, CD117, TLE-1, cytokeratin 7, cytokeratin 20, and p63. The lack of osteoid matrix and the diffuse and strong immunoreactivity with cytokeratin were interpreted as most consistent with metastatic sarcomatoid carcinoma.

A positron emission tomography (PET/CT) was performed to complete the search for an occult primary site of an elusive carcinoma, but this was otherwise negative except in the right distal femur. Limited activity seen in the bilateral lung hilum was not felt to be indicative of obvious primary or metastatic disease, a reactive process being favored.

Medical and radiation oncology consultations were obtained. CT-guided needle biopsy of four mediastinal nodes identified as being enlarged on PET-CT showed reactive disease. The patient sought a second opinion at another institution, where the pathology review concurred with the diagnosis of metastatic sarcomatoid carcinoma. With the diagnosis of solitary metastatic sarcomatoid carcinoma of unknown primary, the patient underwent external beam radiation therapy (EBRT) to the right distal femur, fifteen cycles of 250 cGy for a total of 3750 cGy.

The clinical response to EBRT was poor, with worsening overall pain, nighttime awakening pain, antalgia with slightest weight-bearing, decreased range of motion, increased swelling, and overall poor function of the knee. In view of the lack of primary site, the poor prognosis and limited treatment options for metastatic carcinoma of unknown primary, and the possibility of a primary bone tumor after microscopic examination of the entire tumor, surgical resection was performed.

**Figure 4:**
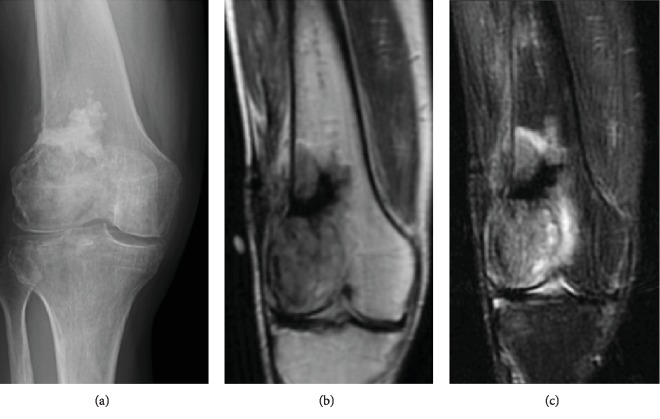
(a) Post-EBRT X-ray of the involved distal femur shows some periosteal reaction developing. Intramedullary sclerotic changes are seen reflecting the introduction of bone cement after the initial biopsy. (b) Postradiotherapy MRI demonstrating the lesion has increased in size since the previous examination now measuring 8.5 cm in the cephalocaudal dimension and 4.5 cm in the transverse dimension with evidence for postoperative change within the lesion. Patchy areas of increased signal within the medullary space of the mid to distal femur as well as the proximal tibia and patella most likely are related to postradiation change. The focal prominent region of decreased signal within the lesion corresponds to interval biopsy, curettage, and cementation. (c) Dark heterogeneous signal on T1W and brighter signal intensity on T2W sequences.

Repeat X-rays showed some demineralization, and repeat MRI (Figure 4) showed some progression in the size of the tumor. While still intraosseous and involving the lateral femoral condyle, it had progressed medially past the midline. Wide resection of the distal femur with megaprosthesis reconstruction was performed.

**Figure 5:**
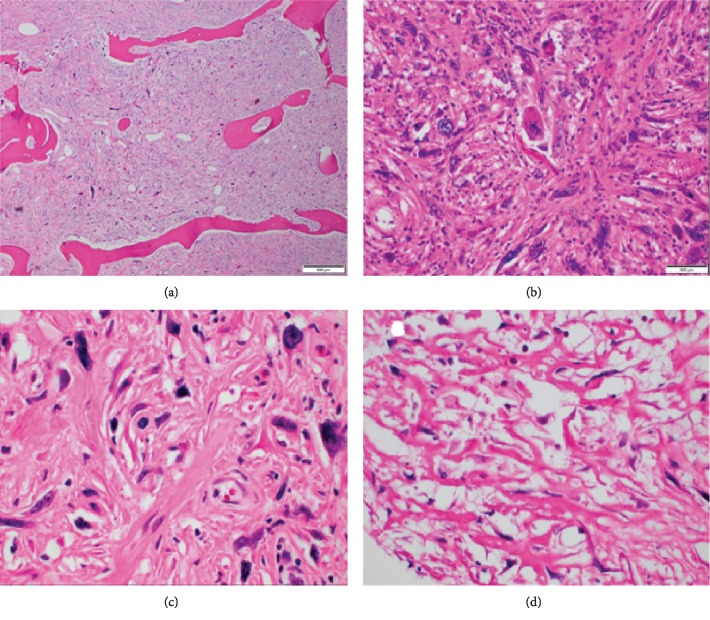
(a–d) Routine histologic sections of the postresection distal femur showing high-grade spindle cell sarcoma with marked nuclear pleomorphism, active mitosis, and areas of necrosis. The neoplasm is poorly differentiated but with focal areas of osteoid matrix. (a) Low power view of tumor permeating cancellous lamellar bone (H&E stain 10x). (b) Undifferentiated pleomorphic component (H&E stain, 40x). (c) Pleomorphic malignant cells and osteoid matrix (H&E stain, 40x). (d) Osteoid matrix with scattered malignant cells (H&E stain, 20x).

**Figure 6:**
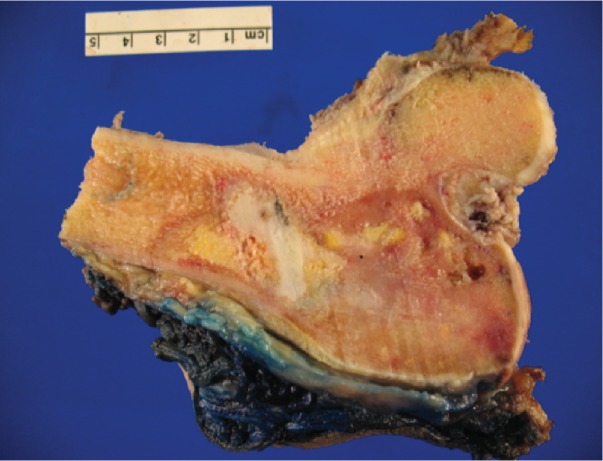
Resection specimen. Tumor replacing cancellous bone and invading cortex. Cement (white area) from needle biopsy procedure.

**Figure 7:**
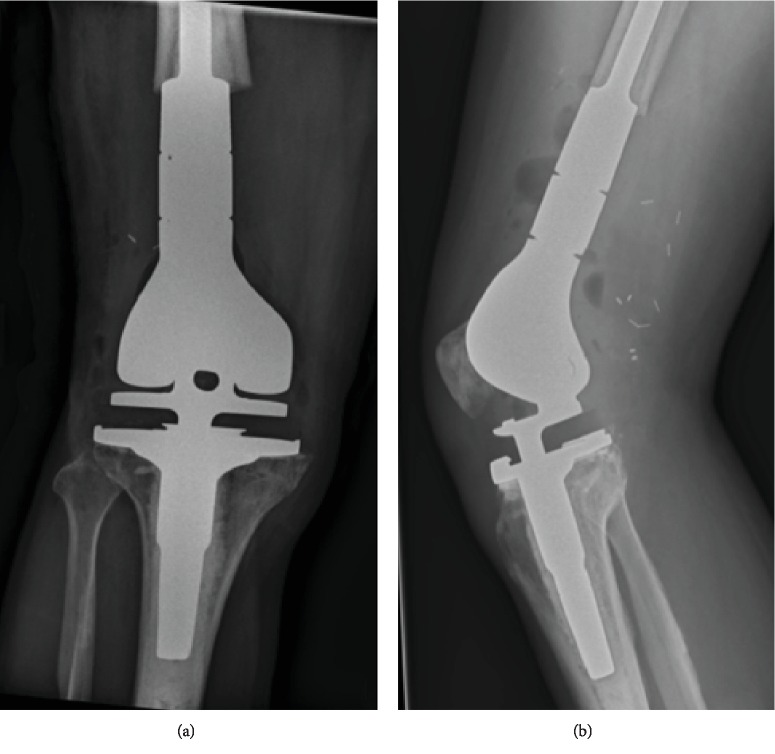
Latest follow-up XR of the distal femur reconstruction, two views. Status postresection of the distal femur with megaprosthesis replacement that is incorporated in a right knee arthroplasty.

**Figure 8:**
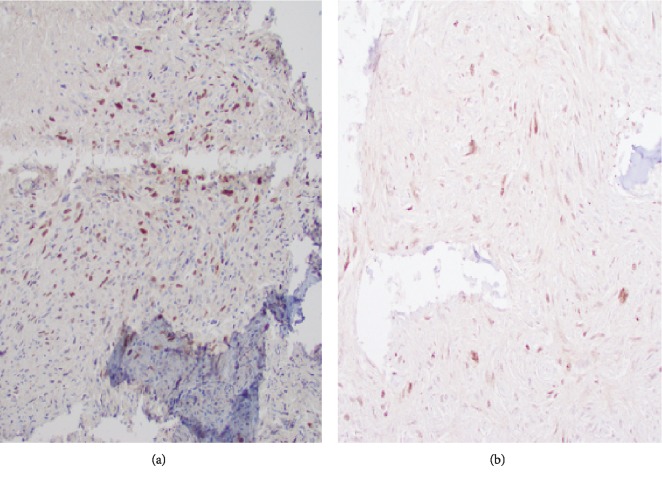
SATB-2 immunostain shows patchy but strong nuclear positivity in the tumor cells in both the prechemotherapy biopsy tissue (a) and postchemotherapy resection specimen (b).

The distal femoral resection specimen showed conventional osteosarcoma (Figure 5). The gross specimen consisted of 12.4 × 8.8 × 7.8 cm segment of the distal femur, which also included the soft tissues and skin from the needle biopsy site scar. The cut surface showed an 8.6 × 5.2 × 4.3 cm ill-defined tumor replacing the trabecular bone of the metaphysis and epiphysis. The tumor invaded the cortex of the lateral epicondyle without cortical breakthrough and extended to within 2.8 cm from the proximal resection margin. A 3.2 × 1.0 cm area of bone cement from the prior needle biopsy was identified in the diaphysis. Histologic sections (Figure 6) show a high-grade spindle cell sarcoma with marked cellular pleomorphism, extensive necrosis, and numerous mitotic figures replacing the marrow and encasing preexisting trabecular or cancellous bone. Focal osteoid matrix was identified. Immunohistochemistry also showed strong tumor cell reactivity for vimentin and cytokeratin CAM 5.2, weak and focal reactivity with cytokeratin AE1/AE3, and lack of reactivity with epithelial membrane antigen, MDM2, and CDK4. Despite the strong cytokeratin immunoreactivity, the overall morphological features were those of a conventional (fibroblastic) osteosarcoma. The patient was scheduled for chemotherapy with cisplatin and adriamycin for a minimum of 6 cycles; however, he decided not to proceed with chemotherapy due to the concern of side effects. Follow-up at 6 weeks showed that he had excellent pain relief, close to full active range of motion, and was ambulating without a cane (Figure 7). He has remained disease-free without evidence of recurrent osteosarcoma through his latest follow-up at 8 months. Ultimately, both the prechemotherapy biopsy tissue and the postchemotherapy resection specimen were tested by immunohistochemistry for SATB-2, and both specimens showed patchy but strong nuclear tumor cell positivity, confirming the diagnosis of osteosarcoma (Figure 8).

## 3. Discussion

Epithelial differentiation, characterized by cytokeratin positivity on immunohistochemical staining, is typical of several epithelial sarcomas such as epithelioid and synovial sarcoma [13]. However, it is rare for osteosarcoma to show an intense and diffuse reaction to cytokeratin on histochemical analysis, normally seen in epithelial tumors [3]. While the most extensive expression of cytokeratin in osteosarcomas is found in tumors with an epithelioid cell component, intense immunoreaction to cytokeratin can also be seen in osteosarcomas without an epithelioid component or appearance [4].

Epithelial features previously reported in osteosarcoma include keratin pearl formation, glandular/rosette-like features, epithelial membrane antigen (EMA), cytokeratin 8 and 18 (detected by antibody CAM 5.2), and cytokeratin AE1/AE3 immunoexpression [4–12]. However, as in our case, there has only been one previous report which demonstrated positive immunohistochemical reactivity for cytokeratin and lack of reactivity with EMA [4].

Histologic distinction between metastatic sarcomatoid carcinoma and cytokeratin-positive osteosarcoma with extensive epithelioid cell proliferation is difficult [1, 4]. The initial pathology sections in our case revealed a lack of osteoid matrix along with diffuse and strong immunoreactivity with cytokeratin and vimentin. The tumor was also positive, albeit focally and weakly, for cytokeratin AE1/AE3 and GATA-3 (a relatively specific marker of urothelial and breast carcinoma). However, while GATA-3 was positive, the reactivity was weak and focal and two other urothelial markers (p63 and CK20) were negative making a metastatic bladder tumor less likely. Given these results, along with the fact that metastases are far more frequent than primary sarcomas in adults, it initially seemed apparent that our patient had metastatic sarcomatoid carcinoma. Further, the SATB-2 (special AT-rich sequence binding protein) antibody for osteosarcoma was not initially available in our lab. Additionally, although SATB-2 expression is highly sensitive for mesenchymal tumors with osteoblastic differentiation, with sensitivity and specificity for osteosarcoma being 90.4% and 95.3%, respectively [14], SATB-2 is also commonly expressed in colorectal tumors [15], some ovarian tumors [16], and rarely upper gastrointestinal and lung tumors with enteric differentiation.

While subsequent histological evaluation of the resected specimen showed evidence of osteosarcoma, consideration might be given to two other possibilities if there was no SATB-2 immunoexpression evidence. First, the malignant osteoid component may have been reactive bone formation as a result of the radiation therapy our patient received. Hence, it may have been that the disease was actually metastatic in nature and that bone formation occurred after receiving EBRT. Second, although extremely rare, de novo osteoid and bone formation has been reported in the literature in metastatic carcinoma [2], as well as in melanoma [17] and colon cancer [18]. However, had his disease been metastatic in nature, it would have been likely that metastases to other sites had appeared at that point or subsequently.

Moreover, metastatic sarcomatoid carcinoma of unknown primary is extremely rare as metastases from sarcomatoid carcinoma are almost invariably associated with identifiable large primary tumors from a variety of organs, such the bladder, kidney, lung, and pancreas, among the most common. On the contrary, our patient remained disease-free since resection and responded well once appropriate treatment was given.

The exact mechanism or pathogenesis of how biphenotypic malignancies forge their existence is not entirely clear. Several hypotheses have been suggested, but the most plausible of them is that of the uncommitted, multipotential stem cell as the cell of origin [8]. This suggests that a primitive mesenchymal cell can acquire the morphology of an epithelial cell and express markers specific to epithelial cells like EMA and cytokeratins.

## 4. Conclusion

Although rare, it is possible for osteosarcoma to show intense and diffuse positivity for epithelial markers such as cytokeratin on immunohistochemical analysis. In distinguishing cytokeratin-positive osteosarcoma from metastatic sarcomatoid carcinoma, the most important finding on histological studies, which was present on resection pathology in our case, is an osteoid matrix or neoplastic bone formation [2]. Furthermore, history of cancer and the existence or absence of other cancers as well as integration with radiologic data may provide useful information. In addition to being aware of common sensitive markers for osteosarcoma such as SATB-2, clinicians should be aware of the possibility of cytokeratin-positive osteosarcoma so that the correct diagnosis can be made and the disease can be appropriately managed.
